# Caffeine reduces oxidative stress to protect against hyperoxia-induced lung injury via the adenosine A2A receptor/cAMP/PKA/Src/ERK1/2/p38MAPK pathway

**DOI:** 10.1080/13510002.2022.2143114

**Published:** 2022-11-10

**Authors:** Xijuan Wang, Shuai Lv, Jianwei Sun, Meihui Zhang, Lei Zhang, Yan Sun, Ziyan Zhao, Dandan Wang, Xinjing Zhao, Jiajie Zhang

**Affiliations:** a Department of Paediatrics, Henan Provincial People’s Hospital, Zhengzhou University People’s Hospital, Henan University People’s Hospital, Zhengzhou, People’s Republic of China; b Department of Gastroenterology, The First Affiliated Hospital of Zhengzhou University, Zhengzhou, People’s Republic of China

**Keywords:** Caffeine, apoptosis, A2AR, cAMP, PKA, ROS, oxidative stress, bronchopulmonary dysplasia

## Abstract

**Objectives:** Caffeine has been shown to reduce the incidence of bronchopulmonary dysplasia (BPD). To investigate the protective mechanism of caffeine in a hyperoxia-based cell model of BPD in vitro.

**Methods:** Type II alveolar epithelial cells (AECs II) were isolated and randomly divided into 6 groups: the normal, hyperoxia, caffeine (50 μM caffeine), antagonist (5 μM ZM241385), agonist (5 μM CGS21680), and DMSO groups. Transfection with siRNA against adenosine A2A receptor (siA2AR) was performed in AECs II.

**Results:** Caffeine alone or in combination with adenosine A2A receptor (A2AR) antagonist inhibited apoptosis, promoted proliferation and reduced oxidative stress (OS). The cyclic adenosine monophosphate (cAMP), protein kinase A (PKA) mRNA, A2AR mRNA and the protein levels of A2AR, phospho-Src, phospho-ERK1/2, phospho-P38 and cleaved caspase-3 were decreased in the caffeine and antagonist groups compared with that in the hyperoxia group. However, the effects of caffeine above were weakened by the A2AR agonist. Knockdown of A2AR showed similar results to caffeine.

**Discussion:** Caffeine can reduce apoptosis, promote proliferation, and alleviate OS in hyperoxia-induced AECs II injury by inhibiting the A2AR/cAMP/PKA/Src/ERK1/2/p38MAPK signaling pathway. A2AR may serve as a promising therapeutic target for BPD in prematurity.

## Introduction

The term ‘bronchopulmonary dysplasia’ (BPD) was first used in Northway et al*.* in 1967 to describe a chronic form of injury to the lungs caused by barotrauma and oxygen injury in preterm infants who require mechanical ventilation [1]. BPD remains the most common chronic lung disease in infants and is associated with increased mortality, respiratory morbidity and increased long-term pulmonary morbidity [2,3]. In the absence of effective interventions, BPD is currently a major therapeutic challenge [4]. BPD has been recognized as a multifactorial disease with numerous prenatal and postnatal components that influence lung development [5], and a large amount of data indicates that oxidative stress (OS) is involved in the development of BPD. OS arises when the production of reactive oxygen species (ROS) overwhelms intrinsic antioxidant defenses. It is becoming increasingly evident that OS is associated with impaired lung development [6]. Oxygen therapy is a crucial treatment for premature infants, but even a very short duration of high oxygen exposure can trigger a cascade of OS, thus leading to oxidative damage [7].

Caffeine is a methylxanthine which has a wide variety of uses including universally used for the prevention or treatment of apnea of prematurity [8,9]. The benefit of early caffeine treatment in preventing BPD was not recognized until a randomized clinical trial showed this unexpected association during secondary data analysis, and this groundbreaking observation dramatically changed clinical practice [10]. Further studies verified this effect, but the mechanisms involved in the efficacy of caffeine use need to be further explored [11].

Adenosine is an endogenous purine nucleoside that is involved in various physiological and pathological functions by activating G protein-coupled receptors. There are four types of adenosine receptors (ARs): A1, A2A, A2B and A3 [12]. Adenosine signaling plays an important role in the pulmonary injury response and participates in the occurrence and development of acute and chronic lung inflammation and chronic lung diseases such as chronic obstructive pulmonary disease, asthma and idiopathic pulmonary fibrosis [13]. Adenosine deaminase (ADA) is an enzyme that is responsible for the degradation of adenosine. Clinical studies have shown that plasma ADA levels in premature infants with BPD are significantly higher than those of non-BPD patients, and there is a positive relationship between ADA levels and the severity of BPD [14]. The loss of ecto-5′-nucleotidase (CD73)-mediated extracellular adenosine production leads to decreased survival, exacerbates pulmonary inflammation and worsens lung development in hyperoxic animal models of BPD [15]. Chen et al. showed that caffeine could protect hyperoxia-induced mouse lungs from oxidative injury by inhibiting the NLRP3 inflammasome and NF-κB pathway [16]. In human organotypic retinal cultures, adenosine A2A receptor (A2AR) blockade could prevent the increase in ROS and the morphological alterations in microglia triggered by elevated pressure [17]. Caffeine is a nonselective A2AR antagonist, and it is not clear whether caffeine can reduce OS to protect against hyperoxia lung injury by antagonizing A2AR.

Normobaric hyperoxia cell culture systems are especially useful model systems for oxidative stress [18]. We examined the underlying mechanisms by which caffeine protects against OS by using a cell culture system *in vitro*. In this study, we first isolated and cultured type II alveolar epithelial cells (AECs II) to establish a hyperoxia-induced AEC II model of BPD and then performed experiments with caffeine treatment, the A2AR antagonist ZM241385, the A2AR agonist CGS21680 and siRNA-A2AR interference to investigate the protective mechanism of caffeine against hyperoxia-induced lung injury *in vitro*.

## Materials and methods

### Animals and reagents

Eight-week-old Sprague–Dawley rats (180∼220 g) were purchased from the Animal Research Center of Zhengzhou University (Zhengzhou, China). The animals were housed in a temperature-controlled environment (25°C ± 2°C) with a 12-h light/dark cycle and free access to food and water. All efforts were made to minimize animal suffering and reduce the number of animals used. The experimental studies were approved by the Laboratory Animal Ethics Committee of Zhengzhou University (ZZU-LAC20210305 [12]). Caffeine (#W222455) was purchased from Sigma-Aldrich (China). The A2AR antagonist ZM241385 and A2AR agonist CGS21680 were purchased from AbMole (China).

### Isolation, purification and culture of AECs II

The isolation process was carried out as previously reported with some modifications [19]. The rats were anesthetized and sacrificed on the 19th day of pregnancy. Under aseptic conditions, 4 fetuses were removed from each pregnant rat, and their lung tissues were separated. Then, the trachea, bronchus and blood vessels and other nonlung tissues were removed, and the lungs were washed and cut into 1 mm^3^ pieces. Then, the tissue blocks were digested with 0.01% trypsin for 15 min, and the cell suspension was collected. After being screened through 100 mesh and centrifuged at 800 rpm for 5 min, the precipitate was digested with 0.1% collagenase for 15 min. After being centrifuged at 1500 rpm for 5 min, the sediment was inoculated in a culture flask and allowed to stand for 40 min, after which the nonadherent cells were aspirated and placed in a culture flask for another 40 min. This process was repeated 3 times. The resultant cells were seeded in Dulbecco's modified Eagle’s medium (DMEM) containing 10% fetal bovine serum (FBS) in the culture flask and incubated for 15–18 h, and then the nonadherent cells were discarded. The remaining cells were isolated AECs II. Finally, AECs II were authenticated by immunofluorescence analysis of SPC-25 (Abcam, ab121395, USA) and vimentin (Abcam, ab137321, USA). AECs II were isolated from 4 fetuses from the same pregnant rat and cultured together, and AECs II isolated from different pregnant rats were cultured separately and subjected to follow-up experiments.

### AECs II treatments

After being identified, AECs II were plated in culture flasks and randomly divided into the following 6 groups: 20%O_2_ (normal) group, 95%O_2_ (hyperoxia) group, caffeine group (95%O_2_ + 50 μM caffeine), antagonist group (95%O_2_ + 50 μM caffeine + 5 μM ZM241385), agonist group (95%O_2 _+ 50 μM caffeine + 5 μM CGS21680), and DMSO group (95%O_2 _+ 50 μM caffeine + equal volume of DMSO). AECs II were exposed to 95% O_2_–5% CO_2_ in a chamber for a pre-determined period of time to establish the hyperoxia injury cell model. The normal group was placed in 95% air–5% CO_2_. All cells were cultured in DMEM containing 10% FBS. Drugs (caffeine, ZM241385, or CGS21680) were added to the medium before exposure, and the doses were determined by preliminary experiments.

### MTT assay

After being treated, AECs II were seeded in triplicate in 48-well plates (1 × 10^5^ cells/mL) and incubated for 0, 12, 24 and 48 h in 95% O_2-_5% CO_2_ or 95% air-5% CO_2_. MTT working solution was added to the wells, and the cells were incubated for another 3–4 h. The medium was removed, and 150 μL of DMSO was added to dissolve the formazan crystals. Cell viability was determined by measuring the absorbance (optical density, OD value) at 490 nm using BIO-RAD iMark Microplate reader.

### Flow cytometric apoptosis assay

After being treated 48 h, AECs II were collected and analyzed using an apoptosis detection kit (BD, 556547, USA). Briefly, after being washed two times, AECs II were resuspended in phosphate-buffered saline (PBS) at a density of 1 × 10^5^ cells/mL, and 10 μL ﬂuorescein isothiocyanate (FITC)-labeled Annexin V and 10 μL propidium iodide (PI) were added. After being incubated for 20 min in the dark, the mixture was analyzed using a FACSCalibur flow cytometer (BD, Accuri C6, USA).

### ROS measurement

ROS generation was measured using chloromethyl-2’,7'-dichlorodihydrofluorescein diacetate (CM-H2DCFDA) staining. Briefly, cells were washed with cold PBS, and then DMEM containing 10 µM CM-H2DCFDA (Invitrogen, Thermo Fisher Scientific) was added. After being incubated for 40 min in the dark, the cells were trypsinized and resuspended in PBS. Finally, a FACSCalibur flow cytometer (BD, Accuri C6, USA) was used to measure the fluorescence at 538 nm.

### OS indices

Cells and tissue samples were homogenized, and the supernatants were used to measure the levels of glutathione (GSH, #A006-2), glutathione oxidized (GSSG, #A061-1), adenosine triphosphate (ATP, #A095), superoxide dismutase (SOD, #A001-3), glutathione peroxidase (GSH-PX, #A005), and catalase (CAT, A007-1). Detection kits were purchased from Nanjing Jiancheng Bioengineering Institute (Nanjing, China). The levels of cyclic adenosine monophosphate (cAMP) were measured by a cAMP-specific CLIA kit (MyBioSource, #MBS2532806, China). The procedure was performed according to the manufacturer’s instructions.

### Quantitative real-time PCR (qPCR)

Total RNA was extracted using TRIzol reagent (Invitrogen, USA). Five hundred nanograms of RNA from each sample were reverse-transcribed into cDNA using a cDNA Synthesis Kit (Takara, Dalian, China). qPCR was performed using SYBR Green and a LightCycler 480 detection system (Roche Diagnostics, IN, USA). The qPCR primers are shown in Table 1. The data were normalized to β-actin expression levels and calculated using the 2^–ΔΔ*Ct*^ formula.

**Table 1.** The primer sequences used in qPCR.

Genes name	Primer name	5′−3′	Accession number
A2AR	A2AR-F	CCATGCTGGGCTGGAACA	NM_001357942
	A2AR-R	CGTTCGTGTTACTGCCCCTTC	
PKA	PKA-F	GCTGGCTTTGATTTACGG	NM_013181
	PKA-R	GATGTTTCGCTTGAGGATA	
MAPK	MAPK-F	GCATCATGGCTGAGCTGTTG	NM_031020
	MAPK-R	GTCCCCGTCAGACGCATTAT	
β-actin	β-actin-F	AGATGACCCAGATCATGTTTGAGA	NM_031144
	β-actin-R	TCCGGAGTCCATCACAATGC	

### Western blotting

The samples were lysed, and then the total protein level was quantified by a BCA protein assay kit (Beyotime, P0010, China). Then, the total proteins were resolved by 12% sodium dodecyl sulfate–polyacrylamide gel electrophoresis and transferred onto a polyvinylidene difluoride membrane (Millipore, Germany). After being blocked with 5% nonfat milk at 37°C for 1 h, the membranes were probed with anti-A2AR (Abcam, ab79714, USA), anti-p-Src (Abcam, ab185617, USA), anti-phospho-extracellular signal-regulated kinase-1/2 (anti-p-ERK1/2) (CST, #4370, USA), anti-phospho-P38 mitogen-activated protein kinases (anti-p-p38 MAPK) (Abcam, ab4822, USA), anti-cleaved caspase-3 (Bioss, bsm-33199M, China), or anti-β-actin (CST, #3700, USA) antibodies at 37°C for 1 h. Then, the membranes were washed three times and incubated with horseradish peroxidase-conjugated secondary antibodies for 1 h at room temperature. Next, the membranes were treated with an ECL solution (Millipore, Darmstadt, Germany) and visualized using ImageJ. Protein levels were assessed as the ratio of the gray value of each protein to that of the corresponding β-actin.

### Knockdown of A2AR

A2AR-siRNA (5′-GCCCATGAATTACATGGTTTA-3′) and its negative control (5′-UUUGUACUACACAAAAGUACUG-3′) were synthesized and purchased from GenePharm (Shanghai, China). AECs II were harvested during log phase and plated in 6-well plates. A2AR-siRNA and its negative control were transfected into AECs II using Lipofectamine 2000 (Invitrogen, Carlsbad, CA, USA) according to the manufacturer’s protocol. The transfection efficiency was measured by qPCR and Western blotting. After being successfully transfected, the cells were divided into the following three subgroups: 20%O_2_ group (95% air-5% CO_2_ for 24 h); 95%O_2_ group (95% O_2_-5% CO_2_ for 24 h); and caffeine group (95% O_2_-5% CO_2 _+ 50 μM caffeine for 24 h). After being treated, AECs II were collected, and ROS, apoptosis, proliferation, OS indices and related proteins were measured.

### Statistical analysis

The data were statistically analyzed and graphed using GraphPad Prism 5 (GraphPad Software, USA). All results are presented as the mean values ± standard deviations (SD). Statistically significant differences between groups were determined by Student’s *t*-test. Multiple comparisons were made among ≥3 groups using 1-way ANOVA followed by the Bonferroni post hoc test. The nonparametric Mann–Whitney U test was used if the data were not normally distributed. *P < 0.05 and **P < 0.01 were considered statistically significant.

## Results

### Caffeine inhibits apoptosis and promotes proliferation in a hyperoxia-induced AECs II model

**Figure 1.**
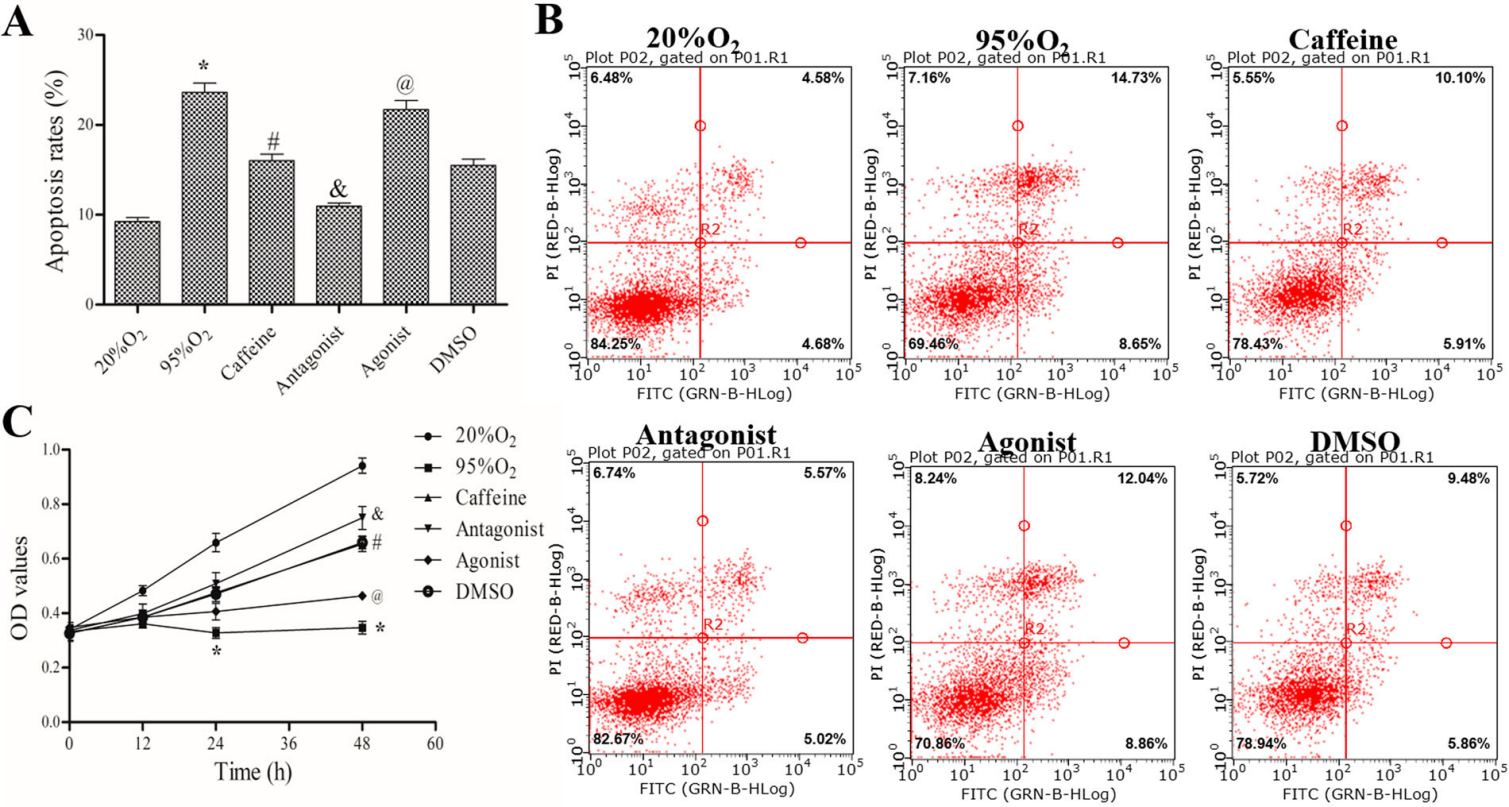
Caffeine inhibits apoptosis and promotes AECs II proliferation. (A and B) A FACSCalibur flow cytometer was used to examine AECs II apoptosis in each group. (C) MTT assays were used to assess cell viability. The data are presented as the mean ± SD (*n* = 3). Compared to the 20%O_2_ group, *P < 0.05. Compared to the 95%O_2_ group, ^#^*P *< 0.05. Compared to the caffeine and DMSO groups, ^&^*P *< 0.05 and ^@^*P *< 0.05.

Immunofluorescence analysis showed that the percentage of SPC-25-positive cells is more than 97%, and the percentage of vimentin-negative cells is more than 96%, indicating we successfully obtained AECs II (Supplemental Figure 1). As shown in Figure 1(A,B), after high oxygen exposure (95% O_2_–5% CO_2_), the apoptosis rate was increased significantly in the 95%O_2_ group compared to that in 20%O_2_ group (*P *< 0.05). Caffeine alone or in combination with the A2AR antagonist ZM241385 markedly inhibited hyperoxia-induced AECs II apoptosis (*P *< 0.05), while the apoptosis of AECs II in agonist group (95%O_2_ + 50μM caffeine + 5μM CGS21680) was increased compared to that in the caffeine and DMSO groups (*P *< 0.05).

With the same starting cell numbers, the OD values were the same in all groups. After different treatments for the same time, the different OD values indicated differences in cell viability. Compared with that in the 20%O_2_ group, when AECs II were treated with 95%O_2_ for 48 h, the OD value was reduced significantly (*P *< 0.05), indicating that hyperoxia can inhibit the proliferation of AECs II. Similarly, after caffeine alone or in combination with ZM241385 treatment, the OD values were increased compared with those in the 95%O_2_ group. However, the addition of the A2AR agonist CGS21680 greatly counteracted the protective effect of caffeine (Figure 1(C)). Flow cytometry and MTT assays indicated that caffeine inhibited apoptosis and promote proliferation in the hyperoxia-induced AECs II model.

### Caffeine inhibits OS in a hyperoxia-induced AECs II model

**Figure 2.**
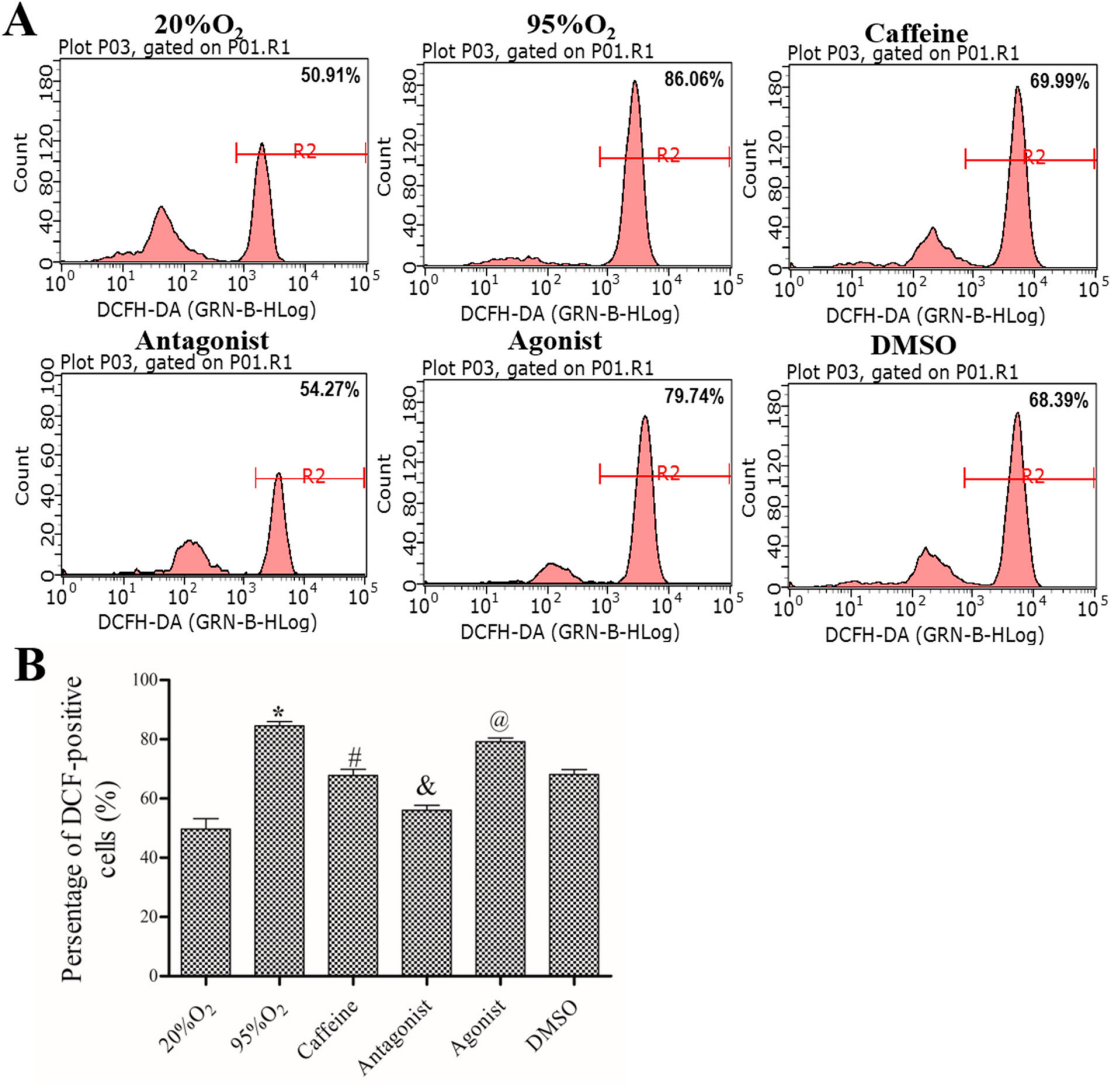
Caffeine inhibits ROS production. (A) A FACSCalibur flow cytometer was used to examine ROS production in each group. (B) Statistical analysis of ROS production. The data are presented as the mean ± SD (*n* = 3). Compared to the 20%O_2_ group, *P < 0.05. Compared to the 95%O_2_ group, ^#^*P *< 0.05. Compared to the caffeine and DMSO groups, ^&^*P *< 0.05 and ^@^*P *< 0.05.

After the different treatments, a FACSCalibur flow cytometer was used to measure ROS production. As shown in Figure 2, the percentage of DCF-positive cells was increased in 95%O_2_ group compared to 20%O_2_ group (*P *< 0.05), and it was decreased after treatments with caffeine alone or a combination of caffeine and ZM241385 in caffeine group and antagonist group (*P *< 0.05). In addition, the percentage of DCF-positive cells continued to decline by adding ZM241385, but the inhibition of DCF-positive cells by caffeine was attenuated with CGS21680 shown in agonist group (all *P *< 0.05). Furthermore, the OS indices are shown in Table 2. Compared with those in the 20%O_2_ group, 95%O_2_ decreased the levels of GSH/GSSG ratio, ATP, SOD, GSH-PX and CAT (*P *< 0.05). After caffeine and ZM241385 treatment, the levels of GSH/GSSG ratio, ATP, SOD, GSH-PX and CAT were increased significantly (*P *< 0.05). However, CGS21680 decreased GSH/GSSG ratio, ATP, SOD, GSH-PX and CAT levels compared with those in the caffeine and DMSO groups (*P *< 0.05). These data indicated that caffeine could inhibit OS in a hyperoxia-induced AECs II model.

**Table 2.** The levels of GSH/GSSG ratio, ATP, SOD, GSH-PX and CAT in AECs II in each group (*n* = 3).

Groups	GSH/GSSG ratio	ATP (µmol/gprot)	SOD (U/mL)	GSH-PX (U/mL)	CAT (U/mgprot)
20%O_2_	6.03 ± 0.13	291.84 ± 23.27	38.13 ± 0.42	146.85 ± 18.87	5.67 ± 0.58
95%O_2_	0.23 ± 0.018*	70.40 ± 13.76*	9.35 ± 1.63*	46.01 ± 5.49*	1.63 ± 0.49*
Caffeine	0.57 ± 0.033^#^	169.93 ± 20.31^#^	13.20 ± 1.57^#^	68.88 ± 7.70^#^	2.28 ± 0.43^#^
Antagonist	2.3 ± 0.13^&^	263.96 ± 24.06^&^	22.71 ± 2.70^&^	95.84 ± 12.85^&^	3.73 ± 0.80^&^
Agonist	0.28 ± 0.028^@^	83.52 ± 12.42^@^	10.90 ± 0.52^@^	52.99 ± 8.78^@^	1.93 ± 0.53^@^
DMSO	0.59 ± 0.034	159.69 ± 18.47	14.00 ± 0.71	66.42 ± 6.18	2.33 ± 0.62

### Caffeine regulates the cAMP/PKA/Src/ERK1/2/p38MAPK pathway in a hyperoxia-induced AECs II model

**Figure 3.**
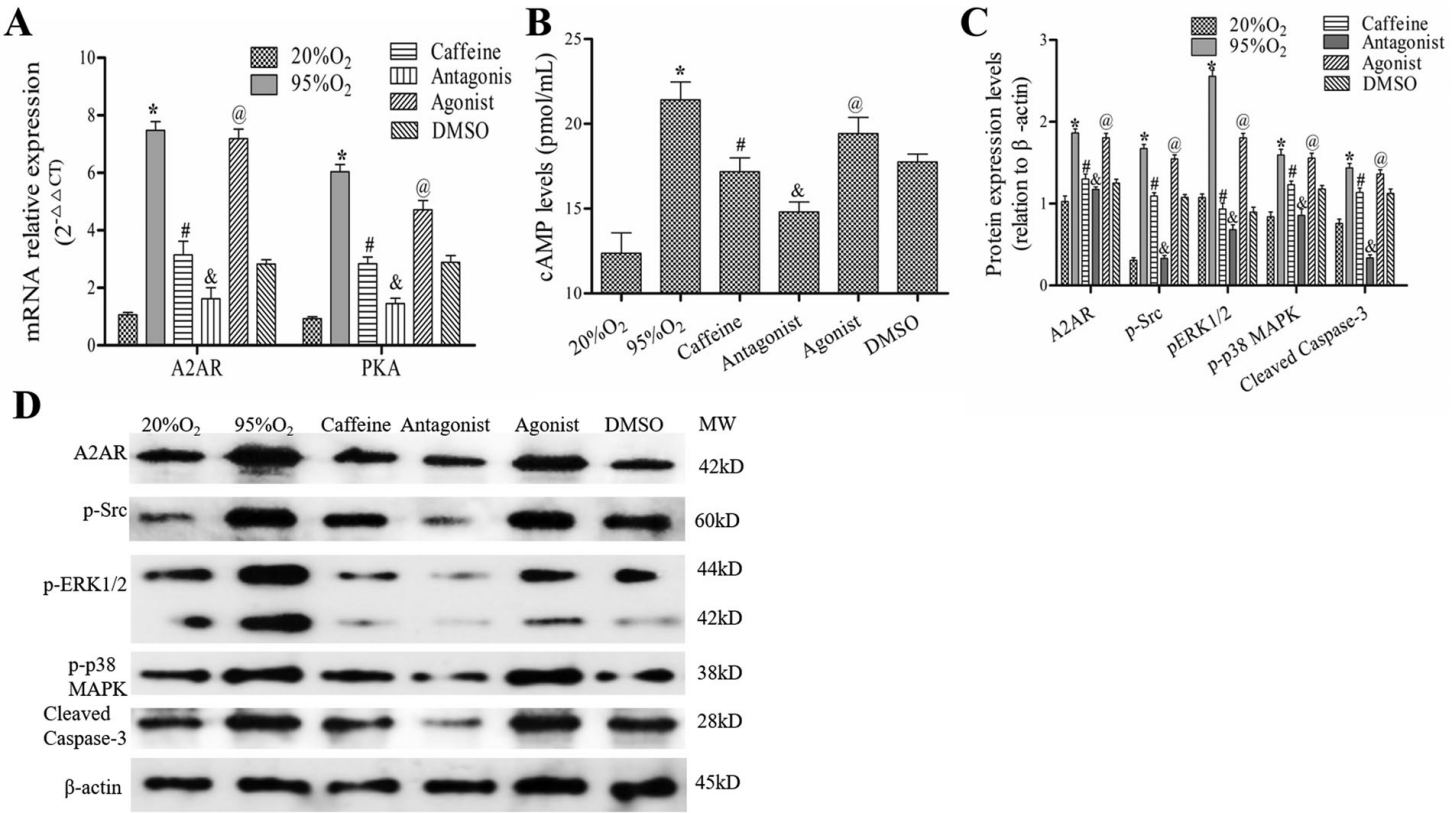
Caffeine regulates the cAMP/PKA/Src/ERK1/2/p38MAPK pathway. (A) qPCR was used to examine the mRNA levels of A2AR and PKA. (B) The levels of cAMP in each group. (C and D) Western blotting was used to examine the protein levels of A2AR, p-Src, p-ERK1/2, p-p38 MAPK and cleaved caspase-3. The data are presented as the mean ± SD (*n* = 3). Compared to the 20%O_2_ group, *P < 0.05. Compared to the 95%O_2_ group, ^#^*P *< 0.05. Compared to the caffeine and DMSO groups, ^&^*P *< 0.05 and ^@^*P *< 0.05.

As shown in Figure 3(A,B), the levels of A2AR mRNA, PKA mRNA, and cAMP were increased markedly after hyperoxia, and they were decreased by caffeine and caffeine/ZM241385 treatment (*P *< 0.05). However, the A2AR agonist CGS21680 increased A2AR mRNA, PKA mRNA and cAMP levels compared with those in the caffeine and DMSO groups (*P *< 0.05). In addition, the protein levels of A2AR, p-Src, p-ERK1/2, p-p38 MAPK and cleaved caspase-3 were decreased significantly in caffeine and antagonist groups compared with those in the 95%O_2_ group (*P *< 0.05). Compared with those in the caffeine and DMSO groups, these levels were markedly increased in the agonist group (Figure 3(C,D)).

These data showed that caffeine could regulate the cAMP/PKA/Src/ERK1/2/p38MAPK pathway in a hyperoxia-induced AECs II model.

### A2AR affects apoptosis and proliferation in a hyperoxia-induced AECs II model

**Figure 4.**
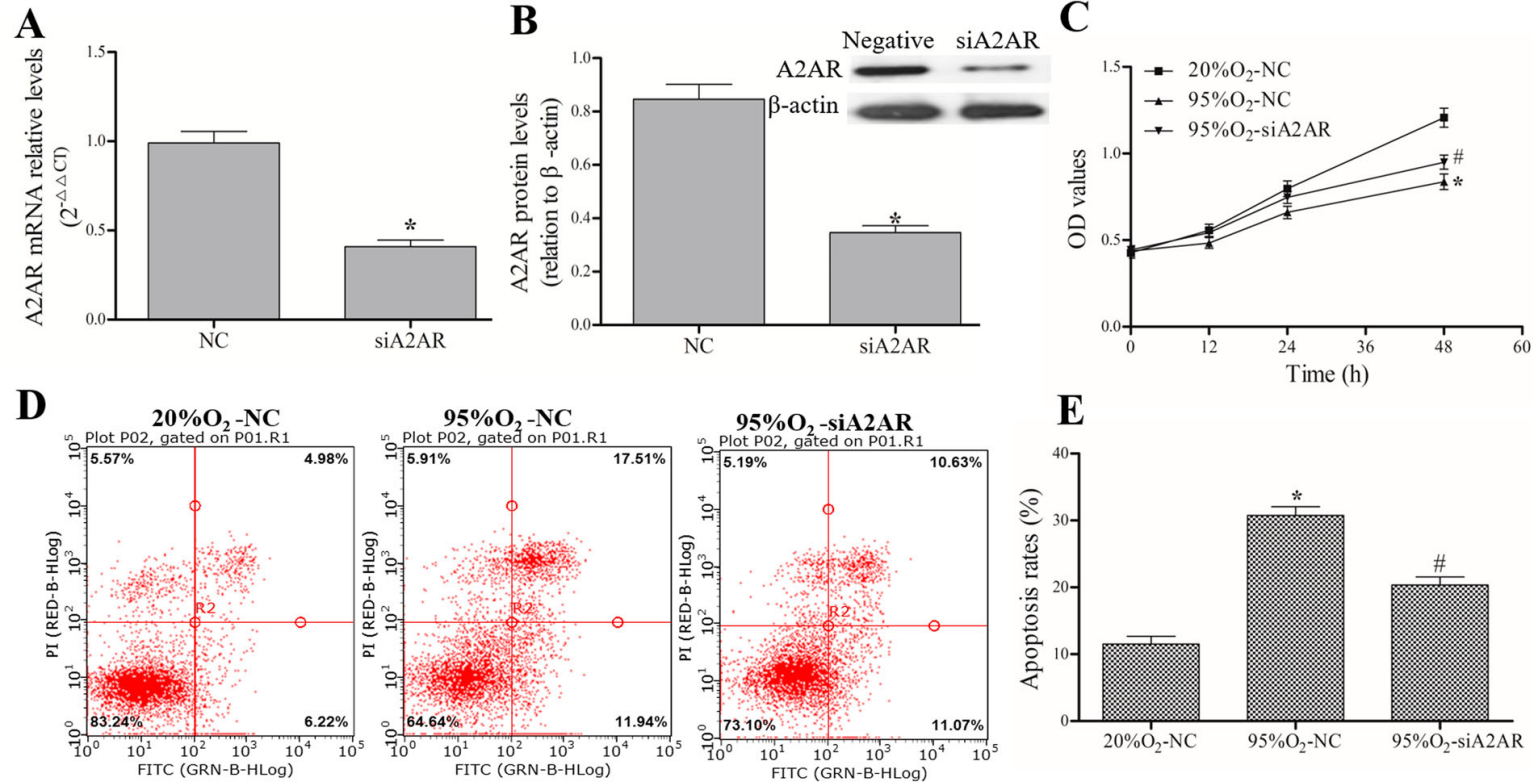
A2AR regulates apoptosis and proliferation in a hyperoxia-induced AECs II model. AECs II were transfected with siA2AR and treated with hyperoxia for 24 h. (A and B) qPCR and Western blotting were used to examine the mRNA and protein levels of A2AR after siA2AR transfection. (C) The MTT assay was used to assess cell viability. (D and E) A FACSCalibur flow cytometer was used to examine apoptosis. The data are presented as the mean ± SD (*n* = 3). Compared to the NC/20%O_2_-NC group, *P < 0.05. Compared to the 95%O_2_-NC group, ^#^*P *< 0.05.

After successful transfection with siA2AR and its negative control (Figure 4(A,B)), AECs II were treated with 95%O_2_ or 20%O_2_. The MTT assay results (Figure 4(C)) showed that 95%O_2_ could inhibit AECs II proliferation, and A2AR knockdown could attenuate the decrease in cell proliferation caused by 95%O_2_. A FACSCalibur flow cytometer (Figure 4(D,E)) was used to show that 95%O_2_ could induce AECs II apoptosis, and A2AR knockdown could attenuate the increase in apoptosis caused by 95%O_2_. The MTT and flow cytometry results indicated that A2AR affect apoptosis and proliferation in the hyperoxia-induced AECs II model.

### A2AR regulates OS and the cAMP/PKA/Src/ERK1/2/p38MAPK pathway in a hyperoxia-induced AECs II model

**Figure 5.**
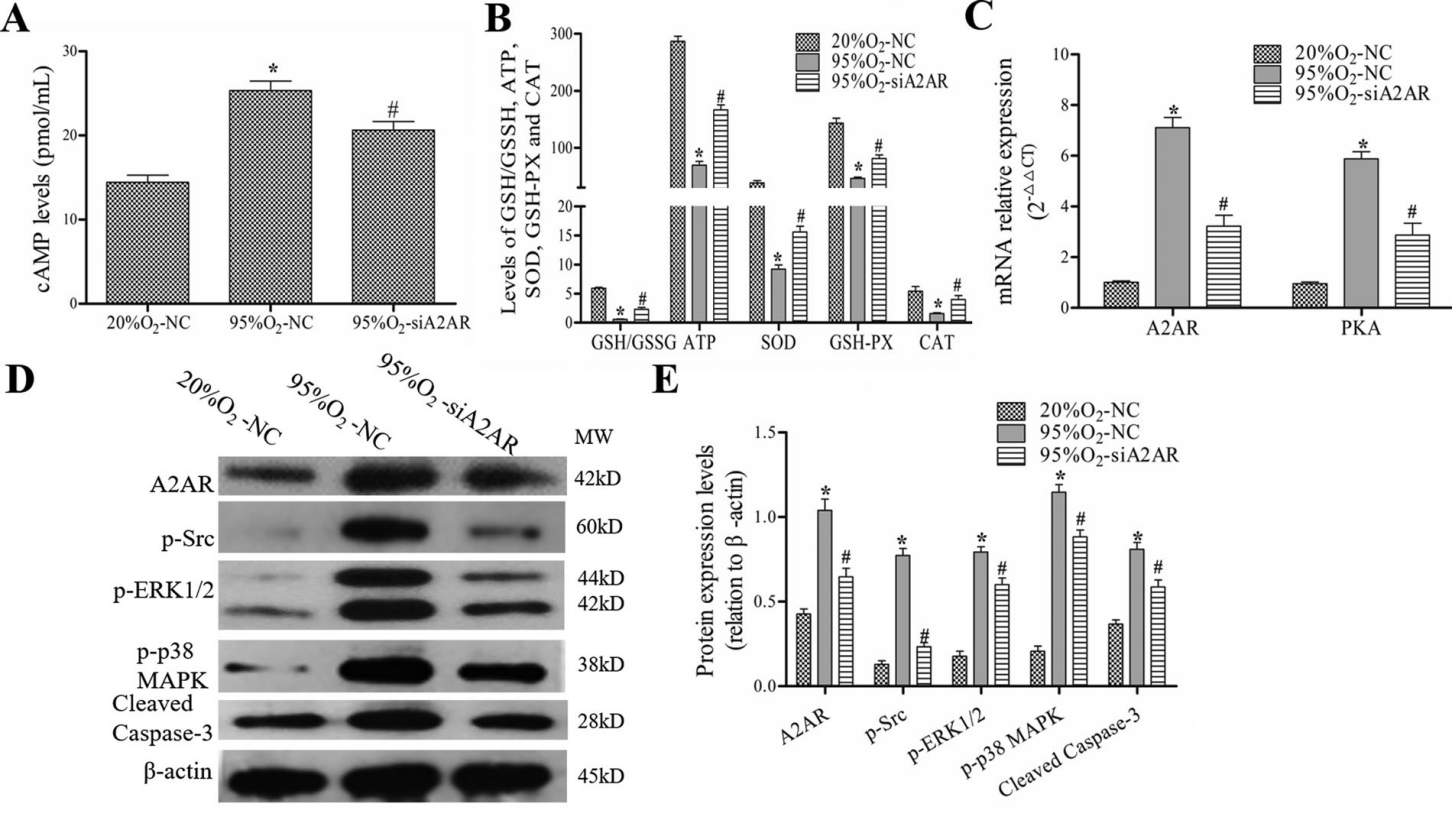
A2AR regulates OS and cAMP/PKA/Src/ERK1/2/p38MAPK pathway-related proteins in a hyperoxia-induced AECs II model. AECs II were transfected with siA2AR and treated with hyperoxia for 24 h. (A and B) The levels of cAMP, GSH/GSSG ratio, ATP, SOD, GSH-PX and CAT. (C) qPCR was used to examine the mRNA levels of A2AR and PKA. (D and E) Western blotting was used to examine the protein levels of A2AR, p-Src, p-ERK1/2, p-p38 MAPK and cleaved caspase-3. The data are presented as the mean ± SD (*n* = 3). Compared to the NC/20%O_2_-NC group, *P < 0.05. Compared to the 95%O_2_-NC group, ^#^*P *< 0.05.

After A2AR knockdown, the levels of cAMP, GSH/GSSG ratio, ATP, SOD, GSH-PX and CAT and the protein levels of A2AR, p-Src, p-ERK1/2, p-p38 MAPK and cleaved caspase-3 were examined. As shown in Figure 5, 95%O_2_ decreased the levels of GSH/GSSG ratio, ATP, SOD, GSH-PX, and CAT, increased cAMP levels, and induced A2AR and PKA mRNA expression and A2AR, p-Src, p-ERK1/2, p-p38 MAPK, and cleaved caspase-3 protein expression. A2AR knockdown reduced cAMP and PKA expression and inhibited the protein expression of A2AR, p-Src, p-ERK1/2, p-p38 MAPK and cleaved caspase-3. These data indicated that A2AR regulate OS and the cAMP/PKA/Src/ERK1/2/p38MAPK pathway.

## Discussion

While advances in neonatal care have resulted in improved survival rates of premature infants, limited progress has been made in reducing the rates of BPD [20], which is not on a lung disease but also a systemic condition with lifelong implications for adult health and quality of life [21,22]. Early initiation of caffeine therapy can not only reduce the incidence of BPD [9] but also improve long-term lung function [23]. A prospective phase 3 clinical trial indicated the early initiation of caffeine has effectively and safely decreased duration of respiratory support used and ICU stay without the development of any complications [24]. A retrospective study showed that high dose caffeine may provide additional benefit in reducing the risk of bronchopulmonary dysplasia and extubation failure, but may also increase the risk of cerebellar hemorrhage and seizures [25]. Another retrospective cohort study indicated preterm infants treated with aminophylline or caffeine had similar respiratory function tests compared with healthy infants at 4–6 years old, but respiratory functions could be affected in the long-term follow-up of premature infants with BPD [26]. It is critical to further explore the mechanisms of caffeine in the pathogenesis of BPD. In an *in vitro* hyperoxia-induced AECs II model, we found that caffeine could inhibit apoptosis and promote the proliferation of primary AECs II, reduce OS, and regulate the cAMP/PKA/Src/ERK1/2/p38MAPK pathway. In addition, A2AR was involved in hyperoxia-induced injury and caffeine-mediated inhibition of OS. In follow-up studies, our team will further verify the pharmacological and molecular mechanisms of caffeine and A2AR in the treatment of BPD in both animal models and clinical studies.

The alveolar epithelium is composed of type I (AECs I) and AECs II. AECs I play active roles in water permeability, gas exchange and the regulation of alveolar fluid homeostasis [27]. AECs II play important roles in the production, secretion, and recycling of lung surfactant [28,29]. AECs II may serve as ‘alveolar stem cells’ and transdifferentiate into AECs I, which are critical for wound repair in BPD [30]. The immaturity of ACEs II is one of the main reasons for atelectasis and respiratory insufficiency after preterm birth. Therefore, AECs II are commonly used to study hyperoxia-induced lung injury. Bai et al. isolated neonatal rat AECs II and investigated the effects of calcitonin gene-related peptide (CGRP) on AECs II exposed to hyperoxia [31]. Duan et al. used rat primary AECs II to investigate the roles of miR-206 and fibronectin 1 in BPD [32]. In this study, we isolated AECs II from fetal rats and treated stimulated with hyperoxia to study the protective effects of caffeine on BPD. In our study, we found that caffeine (50 μM) could inhibit apoptosis and promote the proliferation of AECs II, which was consistent with the study by Tiwari on murine pulmonary epithelial cell lines (A549 and MLE12 cells) [33]. Caffeine (50 μM) was the best concentration selected by preliminary experiments which was also equivalent to the dose (≅10 mg/kg) used clinically in premature neonates. In addition, the A2AR antagonist ZM241385 further strengthen the effects of caffeine, while the A2AR agonist CGS21680 exerted the opposite effect. Subsequent knockout experiments further verified the role of A2AR, suggesting that caffeine may act by antagonizing A2AR.

Premature infants are exposed to increased ROS levels, and they have deficient antioxidant defense systems. There is growing evidence linking early exposure to OS with altered lung development, making the lung more susceptible to a number of diseases that are typical in premature infants, such as respiratory distress syndrome and BPD [34]. OS causes excessive apoptosis in type II pneumocytes and induces an imbalance in mesenchymal–epithelial signaling that leads to the transdifferentiation of pulmonary alveolar lipofibroblasts into myofibroblasts [35,36]. Many studies have attempted to prevent BPD with antioxidant therapies ranging from vitamin and cofactor supplementation to the replacement of antioxidant enzymes that are deficient in preterm infants, such as vitamin E [37], vitamin A and recombinant human CuZn SOD [38,39]. Experimental studies have also shown beneficial effects of antioxidant therapies in preventing and improving BPD [40–42]. Thioredoxin administration markedly attenuated hyperoxia-induced type II cell injury by reducing ROS generation and elevating antioxidant activities [43]. The extrinsic peptide CGRP suppressed hyperoxia-induced apoptosis and OS and ROS production in AECs II [31]. Caffeine has a concentration-specific effect on cell cycle regulation and ROS generation in pulmonary epithelial cell lines under hyperoxic conditions [33]. Caffeine attenuates the activation of cyclooxygenase-2 and markers of apoptosis and protects developing lungs from hyperoxia-induced injury by attenuating endoplasmic reticulum stress [44]. Early caffeine treatment studies showed that caffeine could reduce OS and promote alveolar development in a hyperoxia-based model of BPD in newborn animals [16,45]. We found that caffeine could reduce ROS production and modify OS, further suggesting the antioxidant effect of caffeine. Furthermore, the results showed that A2AR was involved in this process. Indeed, previous research has indicated that A2AR is strongly associated with OS. The A2AR antagonist ZM241385 was an effective suppressor of microglial proliferation and reactivity, OS and photoreceptor apoptosis in a mouse model of retinal detachment [46].

ARs have different affinities for adenosine: A1 and A2A possess high affinity, while A2A and A3 show relatively lower affinity [47]. In addition, the A1 and A3 subtypes are coupled to Gi proteins and have an inhibitory effect on adenylyl cyclase (AC) activity, while A2A and A2B couple to Gs and increase cAMP levels by stimulating AC [48]. Adenosine signaling is central to the pulmonary injury response and usually plays an anti-inflammatory and tissue-protective role in an acute setting through activation of A2A and A2B, while it possesses proinflammatory and tissue-destructive effects in a chronic setting [49]. Upon stimulation, A2AR activates multiple classical signaling pathways, inducing the production of cAMP, which activates PKA [50]. *In vitro* and *in vivo* experiments confirmed that A2AR was involved in the regulation of ROS production and the phosphorylation of ERK1/2, p38 MAPK and Akt in endothelial cells (ECs). Inhibition or blockade of A2AR protects ECs against acute angiotensin II-induced OS, MAPK activation, and endothelial dysfunction [51,52]. Following brain ischemia reperfusion (IR), the dramatic increase in adenosine activates A2AR to induce further neuronal damage. A2AR antagonists were shown to be efficacious in halting IR injury, suppressing apoptotic pathways (cytochrome c and caspase-3) and inhibiting ERK1/2 phosphorylation, thus reducing the inflammatory and excitotoxic cascades [53]. In an alcoholic liver fibrosis study, caffeine significantly inhibited acetaldehyde-induced HSC-T6 cell activation by distinct A2AR-mediated signaling pathways by inhibiting cAMP-PKA-SRC-ERK1/2 for procollagen type I and via P38 MAPK for procollagen type III [54]. In hyperoxia-induced lung injury model rats, caffeine reduced lung tissue fibrosis by blocking the expression of p38 MAPK, thereby playing a protective role [55]. Additionally, Wang et al. found caffeine significantly enhanced angiogenesis in vitro, administration of caffeine significantly promoted angiogenesis and perfusion as well as activation of endothelial AMPK signaling in the ischemic hindlimb, indicating caffeine promote angiogenesis through cAMP/PKA/AMPK signaling pathway [56]. In this study, caffeine treatment and A2AR inhibition or knockdown inhibited OS and the expression of A2AR, p-Src, p-ERK1/2, p-p38 MAPK, cleaved caspase-3, cAMP and PKA, indicating that caffeine was involved in regulating OS via suppressing the A2AR/cAMP/PKA/Src/ERK1/2/p38MAPK pathway.

There are some limitations in this study. Previous studies have shown that caffeine serves as an antioxidant through the direct elimination of ROS [57]. Our results did not distinguish the direct antioxidant effects of caffeine from those acting through antagonizing A2AR. Nevertheless, our study showed that caffeine acts at least in part by antagonizing A2AR.

## Conclusions

**Figure 6.**
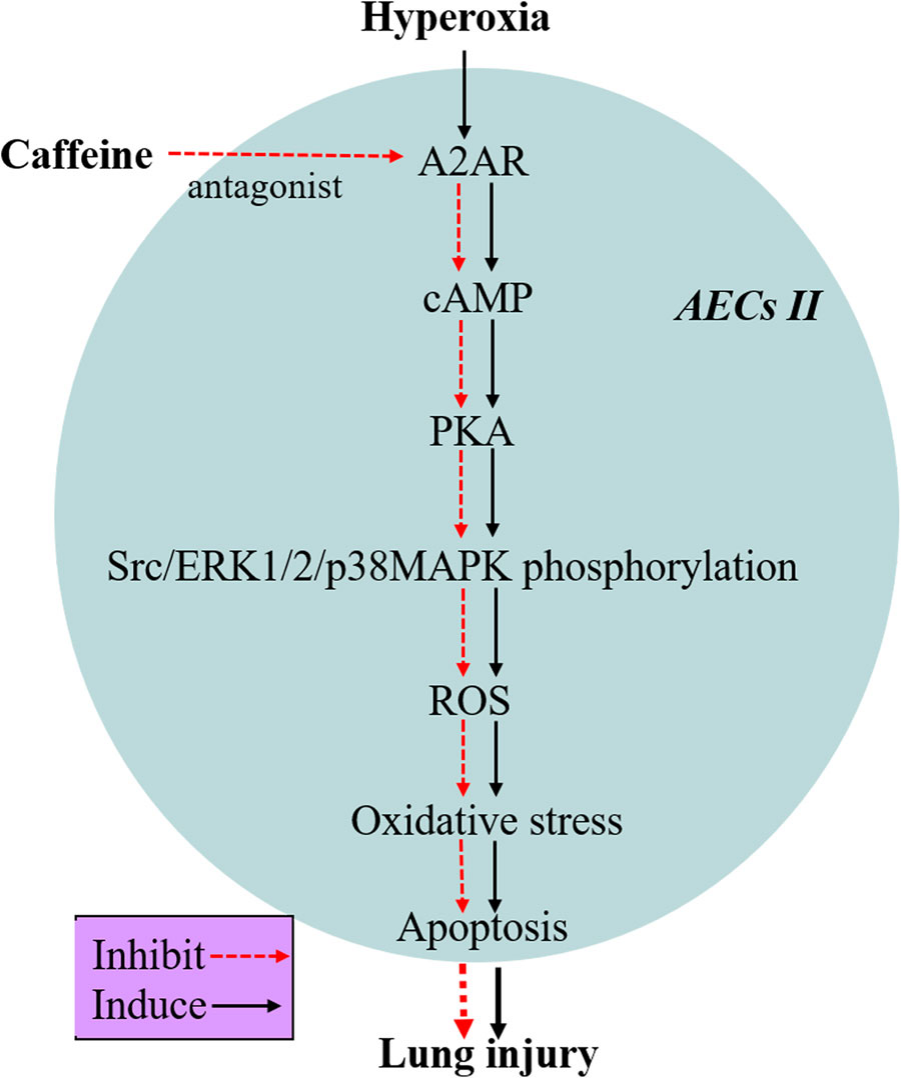
The protective mechanism of caffeine against hyperoxia-induced AECs II injury in vitro.

This study demonstrated that caffeine could inhibit apoptosis, promote the proliferation of AECs II, and reduce OS in hyperoxia-induced AECs II injury, which was demonstrated by inhibiting the A2AR/cAMP/PKA/Src/ERK1/2/p38MAPK signaling pathway (Figure 6). This research has further enriched and clarified the molecular mechanism of caffeine in the treatment of BPD. Furthermore, A2AR may serve as a promising research target in BPD associated with prematurity. In the future, antioxidative therapeutic strategies using caffeine in the treatment of clinical BPD should be the focus of further investigations.

## Supplementary Material

Supplemental Material

## Data Availability

The datasets used and/or analyzed during the present study appear in the submitted article.
