# Supplementary figures and images for: Caffeine reduces oxidative stress to protect against hyperoxia-induced lung injury via the adenosine A2A receptor/cAMP/PKA/Src/ERK1/2/p38MAPK pathway
Source: Redox Rep. 2022 Nov 10;27(1):270–8. doi: 10.1080/13510002.2022.2143114 (PMC9662006; doi:10.1080/13510002.2022.2143114)

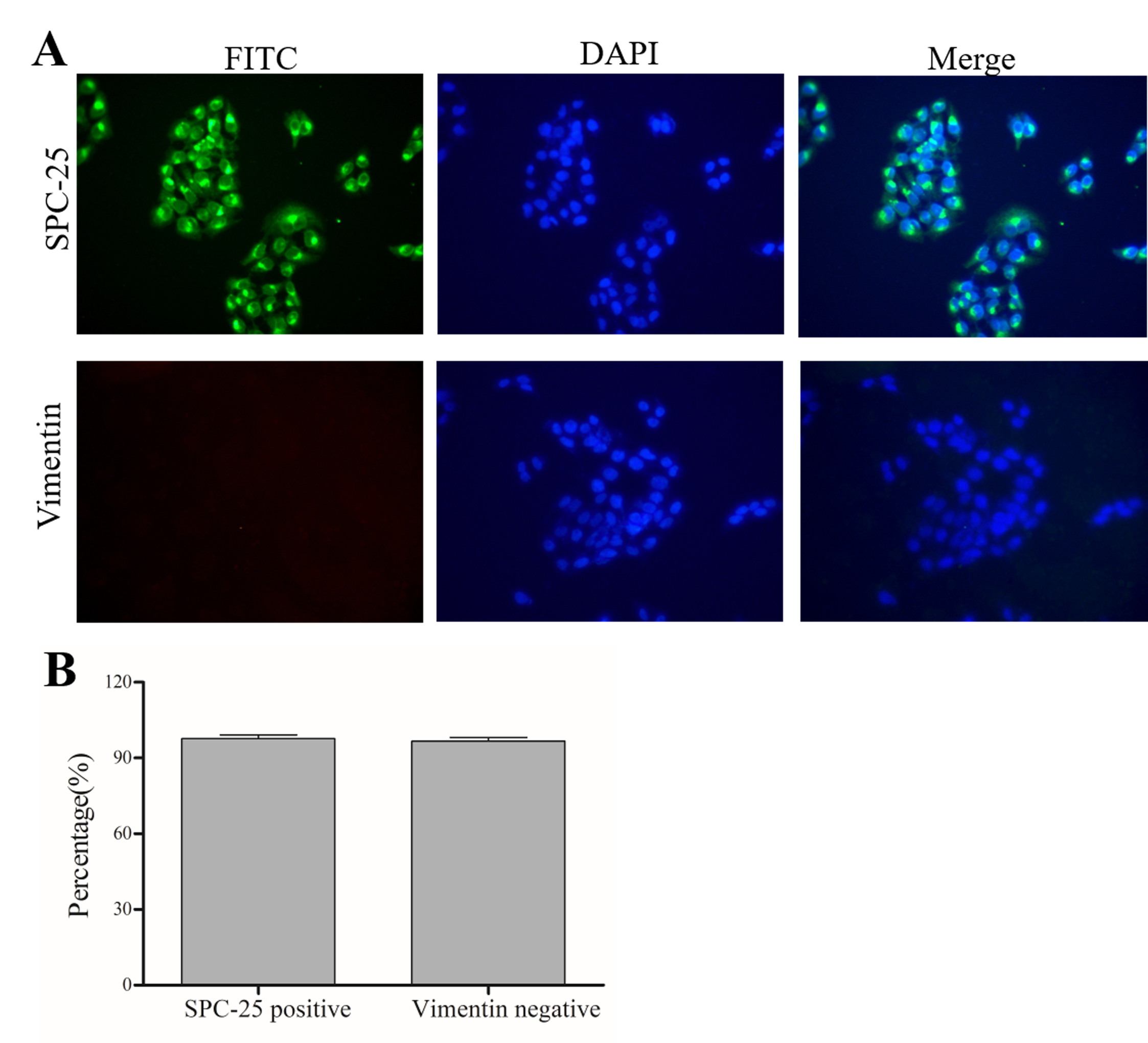

Supplement: Supplemental Material [file YRER_A_2143114_SM9631.tif]
